# Climate Extremes Promote Fatal Co-Infections during Canine Distemper Epidemics in African Lions

**DOI:** 10.1371/journal.pone.0002545

**Published:** 2008-06-25

**Authors:** Linda Munson, Karen A. Terio, Richard Kock, Titus Mlengeya, Melody E. Roelke, Edward Dubovi, Brian Summers, Anthony R. E. Sinclair, Craig Packer

**Affiliations:** 1 Department of Pathology, Microbiology and Immunology, University of California Davis, Davis, California, United States of America; 2 University of Illinois Zoological Pathology Program, Maywood, Illinois, United States of America; 3 African Union International Bureau for Animal Resources, Nairobi, Kenya; 4 Tanzania National Parks, Arusha, Tanzania; 5 Laboratory of Genomic Diversity, SAIC-Frederick, Inc., NCI-Frederick, Frederick, Maryland, United States of America; 6 Department of Population Medicine and Diagnostic Sciences, Cornell University, Ithaca, New York, United States of America; 7 Department of Biomedical Sciences, College of Veterinary Medicine, Cornell University, Ithaca, New York, United States of America; 8 Department of Zoology, University of British Columbia, Vancouver, Canada; 9 Department of Ecology, Evolution, and Behavior, University of Minnesota, St. Paul, Minnesota, United States of America; University of Liverpool, United Kingdom

## Abstract

Extreme climatic conditions may alter historic host-pathogen relationships and synchronize the temporal and spatial convergence of multiple infectious agents, triggering epidemics with far greater mortality than those due to single pathogens. Here we present the first data to clearly illustrate how climate extremes can promote a complex interplay between epidemic and endemic pathogens that are normally tolerated in isolation, but with co-infection, result in catastrophic mortality. A 1994 canine distemper virus (CDV) epidemic in Serengeti lions (*Panthera leo*) coincided with the death of a third of the population, and a second high-mortality CDV epidemic struck the nearby Ngorongoro Crater lion population in 2001. The extent of adult mortalities was unusual for CDV and prompted an investigation into contributing factors. Serological analyses indicated that at least five “silent” CDV epidemics swept through the same two lion populations between 1976 and 2006 without clinical signs or measurable mortality, indicating that CDV was not necessarily fatal. Clinical and pathology findings suggested that hemoparsitism was a major contributing factor during fatal epidemics. Using quantitative real-time PCR, we measured the magnitude of hemoparasite infections in these populations over 22 years and demonstrated significantly higher levels of *Babesia* during the 1994 and 2001 epidemics. *Babesia* levels correlated with mortalities and extent of CDV exposure within prides. The common event preceding the two high mortality CDV outbreaks was extreme drought conditions with wide-spread herbivore die-offs, most notably of Cape buffalo (*Syncerus caffer*). As a consequence of high tick numbers after the resumption of rains and heavy tick infestations of starving buffalo, the lions were infected by unusually high numbers of *Babesia*, infections that were magnified by the immunosuppressive effects of coincident CDV, leading to unprecedented mortality. Such mass mortality events may become increasingly common if climate extremes disrupt historic stable relationships between co-existing pathogens and their susceptible hosts.

## Introduction

Epidemics are usually presumed to be caused by single pathogens, a premise that may in some circumstances be overly simplistic. Temporal and spatial convergence of several infectious agents under environmental conditions that favor their transmission and propagation could create a “perfect storm” of pathogens, resulting in significantly greater mortality, as was suspected in the widespread collapse of honeybee colonies [1]. With global warming, there is considerable concern that ecological patterns of disease will be altered [2], as has occurred with the recent high mortality epidemics in amphibia[3] and corals [4]. Here we present the first data to clearly illustrate how climate extremes can promote a complex interplay between epidemic and endemic pathogens that are normally tolerated in isolation, but with co-infection, result in catastrophic mortality.

## Results

A 1994 canine distemper virus (CDV) epidemic in Serengeti lions (*Panthera leo*) coincided with the death of a third of the population[5], and another high-mortality CDV epidemic struck the nearby Ngorongoro Crater lion population in 2001[6]. The high adult mortality rate during these CDV epidemics was unusual for wildlife populations [7] and prompted our investigation into the ecology of this disease.

**Figure 1:**
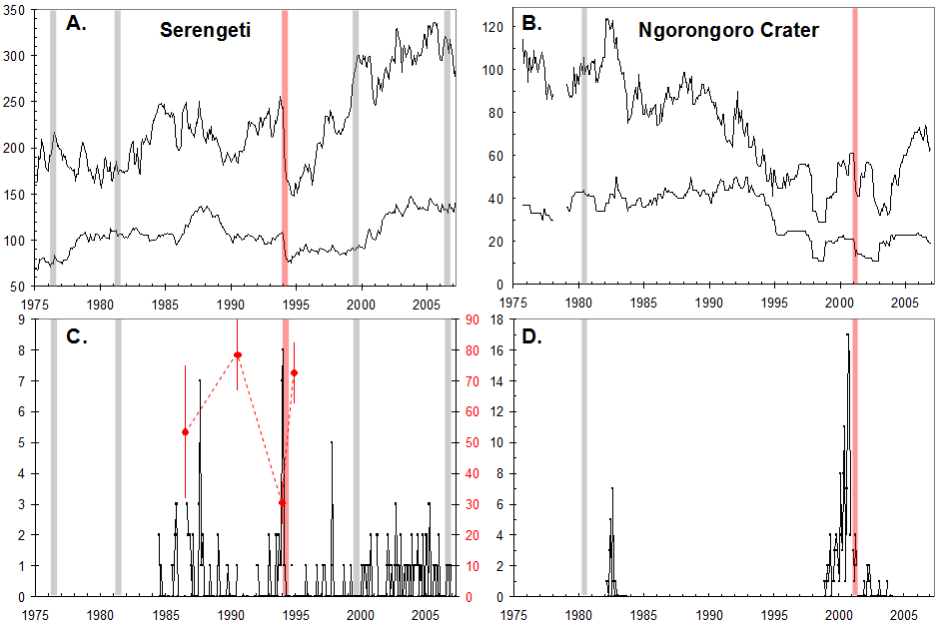
Timing and impact of CDV outbreaks in (A) Serengeti and (B) Ngorongoro Crater. The heavy lines show the number of adults (≥4 yrs of age), and the narrow lines show the total populations. Blue bars indicate likely timing of “silent” outbreaks that were only detected retrospectively by serology; pink bars show fatal outbreaks. Note that serological data are unavailable for the Crater from 1991–2000. (C, D) Number of buffalo carcasses in the diet of the respective lion populations each year and bone marrow fat scores of Serengeti buffalo. Data are restricted to years with comparable levels of search effort. Red circles in the Serengeti (C) show the bone marrow fat scores (% of bone marrow that was fat) of buffalo carcasses. Note: Full-time lion staff was not stationed in the Crater during 1984–98.

The history of CDV exposure in the Serengeti and Ngorongoro lion populations was reconstructed through a retrospective survey. Serological assays of 599 blood samples collected from 510 known-aged lions between July 1984 and January 2007 revealed a repeated pattern of prolonged absence (5–13 yrs in duration) of CDV infection from the Serengeti (as indicated by negative serum titers) punctuated by short periods with widespread (60–95%) CDV exposure. The duration of these epidemics could only be estimated from the two CDV outbreaks with coincident increases in mortality (6–7 mo in the Serengeti study area in 1994 and 10 wks in Ngorongoro in 2001). Fig. 1(A,B) superimposes the timing of each CDV outbreak on the monthly lion population totals, highlighting the two fatal outbreaks in red and illustrating the absence of appreciable mortality during the remaining five outbreaks. The two fatal epidemics occurred under strikingly similar circumstances: severe drought followed by heavy rains. The Serengeti drought of 1993 was the worst in >40 yrs, resulting in extensive ungulate deaths[8], and the Crater drought of 2000 was equally severe[6], [9], suggesting that a climate-associated factor contributed to lion mortalities during CDV exposure.

The Crater epidemic in 2001 provided a unique opportunity to investigate the linkage between severe drought and CDV-related mortality. Ten anesthetized lions were examined over a 5-day period in the 10-wk span when 24 of the 61 Crater lions died. Five of these 10 lions were in poor physical condition and eight had fevers >39° C, as well as enlarged lymph nodes and spleens. Many had skin ulcers associated with *Stomoxys* fly infestations, and some were clinically dehydrated. All 10 lions had macrocytic normochromic anemia with hematocrits ranging from 18–32% [normal lion ranges being 35–45% (International Species Inventory System, Eagan, MN 55121-1170 USA)], total red cell numbers ranging from 3.8–4.8×10^12^/l (average of 4.3×10^12^/l with normal = 7–8×10^12^/l), and hemoglobin ranging from 7–12 g/l (average of 9.3 g/l with normal = 8–12 g/dl), an anemia consistent with ongoing intravascular hemolysis and not CDV, and a dying cub was observed with bloody urine, consistent with hemolysis from hemoparasitism[10]. Together, these findings suggested hemoparasitism was a major contributing factor to morbidity and mortality. Antibodies against CDV were detected in all 10 examined lions, including a 5-mo-old cub (an age when passive immunity usually is undetectable), and high titers (1∶256–1∶3072) were present in five of the lions, providing strong evidence of recent recovery from CDV exposure.

**Figure 2:**
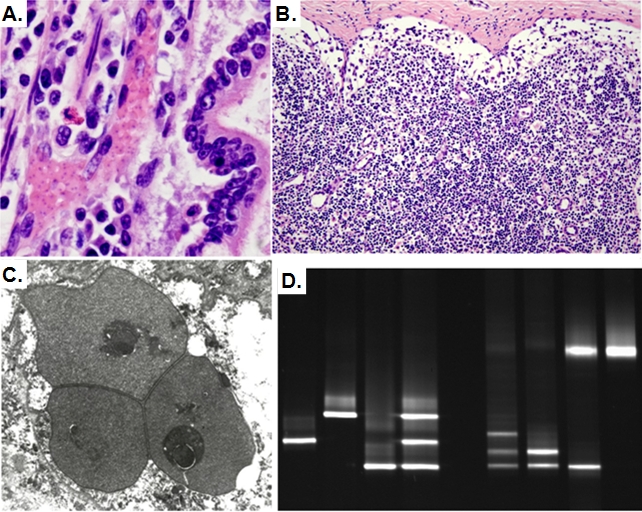
*Babesia* infection in lions from the 2001 epidemic. A) Histopathology of *Babesia* hyperinfection in an adult lion that died during the epidemic. Small intestinal capillaries are occluded by parasitized red blood cells; B) Marked lymphocyte depletion in the lymph node of the same lion, indicating immunosuppression; C) Electron micrograph of intraerythrocytic piroplasms morphologically compatible with *Babesia* sp. in the deceased lion; D) Results of denaturant gradient gel electrophoresis for previously characterized carnivore hemoparasites and hemoparasites amplified by PCR from lion samples that demonstrated mixed infections. Lane 1, *Babesia canis*; lane 2, *Cytauxzoon felis*; lane 3, *B. gibsoni*; lane 4, mixture of *B. canis*, *C. felis*, and *B. gibsoni* isolates in lanes 1–3; lane 5, no DNA-negative control; lane 6, lion with *Babesia sp.* most similar genetically to *B. gibsoni*, *B. felis* and a previously uncharacterized *Babesia sp.*; lane 7, lion with *Babesia sp.* most similar genetically to *B. gibsoni* and *B. felis*; lane 8, lion with hemoparasites most similar genetically to *B. gibsoni* and *Hepatozoon felis*; lane 9, lion with a hemoparasite most similar genetically to *H. felis*.

Although nearly 40% of the population was lost during the epidemic, only two lion carcasses were located for necropsy, an adult found dead and a 5-mo old orphaned cub that was killed by hyenas. The adult suffered from severe systemic babesiosis with low numbers of red blood cells in most organs (suggestive of anemia), sludging of parasitized red blood cells in intestinal capillaries (Fig. 2 A,C), and widespread erythrophagocytosis in the spleen and lymph nodes. The orphaned cub also had widespread babesiosis. Additionally, both lions had marked lymphocyte depletion in lymph nodes (Fig. 2B), suggesting profound immunosuppression consistent with recent CDV infection[7]. Active CDV infection was ruled out as the cause of death in both lions because no viral cytopathic changes, such as syncytia or cellular inclusions, were noted by histopathology, and imunohistochemistry for CDV was negative.

**Figure 3:**
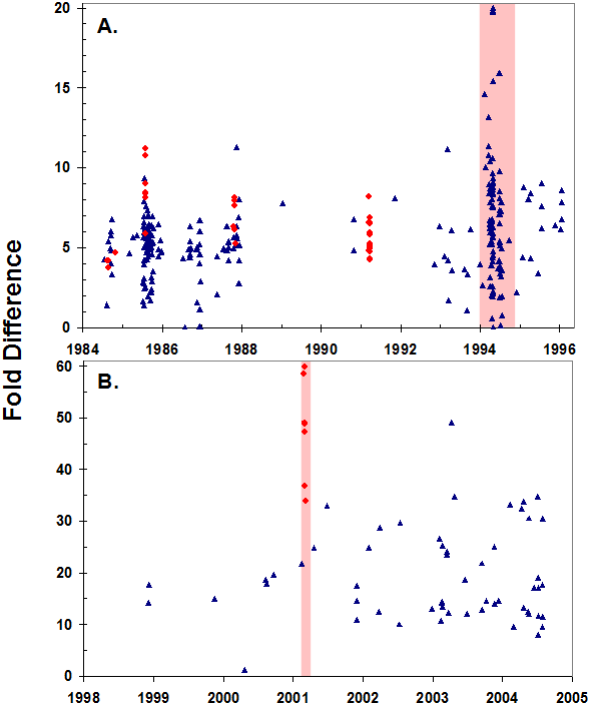
Relative quantity of *Babesia* in lions sampled each year in the Serengeti (blue triangles) and Ngorongoro Crater (red circles) as determined by real-time PCR. Fatal outbreaks are highlighted in light red. Data are separated according to changes in sample preparation affecting levels of PCR product: (A) red blood cell pellets were extracted from all samples collected in 1984–1996, while (B) whole blood was collected for all subsequent samples. The relative quantity of hemoparasite DNA was calculated as average threshold PCR cycle divided by hemoglobin concentration and expressed as the fold difference greater than the sample with the smallest quantity of hemoparasite DNA. Levels of *Babesia* infection were significantly higher during the fatal outbreaks, in the Crater, and in assays performed on whole blood (all three factors significant at P<0.0001 in a multivariate analysis, *n* = 344).

*Babesia* is a tick-borne intraerythrocytic protozoan (hemoparasite) that usually infects African lions at low levels without compromising their health [11], [12]. However in both the 1994 Serengeti and 2001 Crater CDV epidemics, unusually abundant *Babesia* were noted in blood smears collected from lions. To quantify these hemoparasite infections and determine if levels increased during high-mortality CDV epidemics, we measured hemoparasite burdens by quantitative real-time PCR using primers designed to detect *Babesia*, *Theileria*, *Cytauxzoon*, and *Hepatozoon*. We assayed a total of 346 blood samples from 301 individuals collected from 1984–2004, comparing hemoparasite levels during CDV epidemics with typical infection levels in these populations. We found that hemoparasite burdens were significantly higher during the high-mortality 1994 and 2001 lion die-offs (Fig. 3). All of the Crater lions that were found to be lethargic, anemic, and febrile when examined in early 2001 and from which blood was available for PCR (*n* = 6), had hemoparasite levels that were among the highest measured (fold differences of 37–60). Also, the orphaned cub that was killed by hyenas similarly had high hemoparasite levels (fold difference of 34) consistent with the histologic findings of severe babesiosis.

We then investigated whether the increased load of hemoparasites was due to the introduction of novel pathogens during the fatal CDV epidemics. Four species of *Babesia* (most similar to *B. felis*, *B. leo*, *B. gibsoni* and an uncharacterized *Babesia* sp.) and *Hepatozoon felis* (Fig. 2D) were detected in the populations throughout the entire 20 year study period except for *B. leo* (the common *Babesia* of southern African lions [11]) that was only detected in two Serengeti lions sampled in 1985 and 1987. Samples acquired at different times from 32 lions were examined, and infections with different species of *Babesia* were acquired or cleared with no temporal trend. These data indicate that mortality during the fatal CDV epidemics was not associated with an introduced species or greater numbers of co-infecting *Babesia* species. We also found *Hepatozoon* in many lions and compared levels across time. *Hepatozoon* levels were not associated with fatal outbreaks, as might be expected because *Hepatozoan* infections are usually subclinical in lions. To rule out other blood-borne infections, we further tested a subset of 30 lions for infection with *Mycoplasma hemofelis* and *M. hemominutium*, only finding *M. hemominutium*. *Mycoplasma* levels did not parallel hemoparasite levels or increase during lethal CDV epidemics. Similarly, feline immunodeficiency virus (FIV) infection was ruled out as a co-factor because nearly all of the adult Serengeti and Crater lions have been FIV+ each year since at least 1984, and FIV-infection in lions has never been linked with mortality[13], [14]. Together these data indicate that only *Babesia* co-infection correlated with mortality trends.

**Figure 4:**
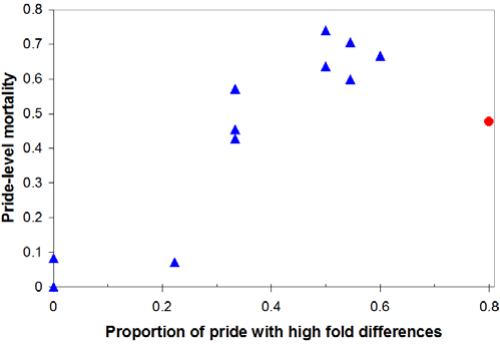
Pride-level effects of hemoparasitemia on mortality rates during the fatal outbreaks in the Crater (red circle) and Serengeti (blue triangles). Prides with a greater proportion of individuals showing high hemoparasitemia also suffered higher mortality rates (P<0.01).

Variations in infection and mortality across lion social groups (prides) implicate an interactive effect of CDV and *Babesia*: prides with higher average CDV titers had a greater proportion of individuals with high levels of *Babesia* (r^2^ = 0.41, n = 11 CDV-infected prides, P<0.05), and prides with the highest levels of *Babesia* suffered the highest mortality rates with many prides suffering >67% mortality (r^2^ = 0.63, n = 12 prides, P<0.01) (Fig. 4). The only Serengeti pride to remain uninfected by CDV in 1994 had only moderate *Babesia* levels and no increase in mortality.

In other species, death of CDV-infected animals has been attributed to overwhelming infections with co-pathogens, such as toxoplasmosis, that result from CDV-induced immunosuppression[7], [15]–[17]. Yet these co-infections have not caused deaths in epidemic proportions as occurred in the lion populations. CDV-induced immunosuppression alone cannot explain the *Babesia* hyperinfection during fatal CDV epidemics, because immunosuppression would also have occurred during silent CDV epidemics. Thus the severity of babesiosis during the high fatality epidemics must be attributable in part to the environmental conditions in 1994 and 2001 that strongly favored the propagation of tick vectors for hemoparasites in the lions.

Tick levels in the Serengeti ecosystem during the 1993–4 and 2001 droughts were at unusually high levels due to the general debility of the herbivores and to pasture management in the Crater[9]. Several black rhinoceros (*Diceros bicornis*) in the Crater died during 2001 due to fulminating babesiosis that was attributed to the extensive tick infestations[9]. While the same climate extremes may have directly affected tick and consequently hemoparasite burdens in the lions, the impact of drought was intensified by its effect on a key prey species, the Cape buffalo. The buffalo were severely nutritionally compromised by the 1994 and 2001 droughts, as evidenced by low bone marrow fat quantification scores (Fig. 1 C, D), and with resumption of the rains, the compromised buffalo developed high tick burdens[18]. Large die-offs of buffalo occurred immediately prior to, during, and following the CDV epidemics, permitting the lions to feed on unprecedented numbers of tick-infested buffalo carcasses (Fig. 1 C,D). Some ixodid ticks, such as *Rhipicephalus appendiculatus*, *Rhipicephalus simus*, and *Amblyomma hebraeum* are shared between wild herbivores and lions [19], [20] and, as potential vectors for *B. felis*, *B. leo*, and *B. gibsoni* [11], [21], [22], may have completed some of the life cycle stages necessary for *Babesia* infectivity on the dying buffalo. In support of that hypothesis, hemoparasite levels were significantly higher in lions that fed on buffalo during the 1994 Serengeti die-off than in lions from prides with low buffalo exposure (P = 0.0011, Mann-Whitney). Among buffalo-eating lions, hemoparasite levels were significantly higher in lions that had been infected with CDV (P = 0.0099), demonstrating the permissive effect of viral immunosuppression. Two prides that were exposed to CDV but did not prey on buffalo, had lower levels of *Babesia* infection and no increased mortality in 1994, evidence that exposure to buffalo influenced disease outcome in lions.

## Discussion

The higher burden of *Babesia* in the Crater than Serengeti lions may have resulted from a variety of ecological and genetic factors. The Crater floor supports a high density of large mammalian herbivores and associated carnivores that can serve as hosts to *Babesia* vectors. Anthropogenic landscape alterations also may have contributed to heavy tick burdens. Grass fire on the Crater floor was actively suppressed from the 1970s until the high tick infestations of 2001[9] (and associated deaths of several rhinoceros[23]) led to a new policy of controlled burning [9]. Genetic homogeneity may also play a role because the Crater lions are highly inbred compared to their Serengeti counterparts [24] and may be more susceptible to parasitic and viral infections [6]. *Stomoxys* flies also flourished in the rains following the Crater drought, causing pruritic skin ulcers on lions similar to those seen during a severe (but undiagnosed) lion die-off in the Crater in 1962[25]. We tested *Stomoxys* flies collected from lions in 2001 and successfully amplified *Babesia* sp. and *Hepatozoon* sp., but could not confirm whether the flies served as additional biological *Babesia* vectors during the epidemic.

Our findings show that high-mortality CDV epidemics coincided with unusually high levels of *Babesia* infection resulting from climatic events that increased the lions' exposure to tick-infested prey. The combination of high tick exposure and CDV-related immunosuppression caused the hemoparasite infections to become fulminating. If extreme weather events become increasingly frequent owing to global climate change, the consequent synchronization of proliferating pathogens or their vectors may cause disease to become a major threat to historically stable populations that had previously coexisted with multiple viral and parasitic pathogens.

## Materials and Methods

### Blood and tissue samples

Blood samples (309 from Serengeti; 37 from Ngorongoro Crater) were collected from African lions (266 in the Serengeti; 35 in Ngorongoro) every year between 1984–2004 except 1988 and 1997. Blood was stored either as whole blood in EDTA or as packed red blood cells and serum. All samples were first preserved in liquid nitrogen before being stored at −70°C. Serum was tested for CDV antibodies by a viral neutralization assay using Onderstepoort CDV strain inoculated onto Vero cells.

Histopathology and immunohistochemistry to detect CDV antigens in tissues were performed by published methods.[5] For electron microscopy, samples were selected from formalin-fixed paraffin-embedded small intestines that had evidence of hemoparasites occluding the small intestinal capillaries by light microscopy. Tissues were excised from the paraffin block, deparaffinized, post-fixed in 2% OsO_4_, embedded in Epon, sectioned at 50 nm, stained with lead citrate and uranyl acetate, and examined on a Phillips 301 electron microscope.

### Quantitative Real-time PCR

Total hemoparasite DNA was quantified from whole blood or red blood cells (RBC) by real-time PCR using generic PCR primers designed to amplify a 246 bp fragment of the 18 s rRNA gene in *Babesia sp.*, *Cytauxzoon sp.*, *Theileria sp.*, and *Hepatozoon sp*. DNA was isolated from blood samples and flies using a commercially available kit (DNeasy Tissue Kit, Qiagen, Valencia CA). To quantify levels of selected hemoparasites, generic PCR primers (Bab1: 5′CAG TTG GGG GCA TTC GTA T 3′ and Bab3: 5′ CTC CCC CCA GAA CCC AAA G 3′) were designed that amplified a 246 bp fragment of the 18 s rRNA gene in known *Babesia sp.*, *Cytauxzoon sp.*, *Theileria sp.*, and *Hepatozoon sp*. Total hemoparasite DNA was quantified by real-time PCR (iCycler RT-PCR Thermocycler, Bio-Rad, Richmond, CA, USA). Reaction mixture (25 µl) contained 2.5 pmol forward and reverse primers and 12.5 µl iQ SYBR Green Supermix (Bio-Rad) used according to manufacturers' directions. PCR conditions were 94°C 5 min; 40 cycles of 94°C for 2 min, 55°C for 30 s, 72°C for 30 s; and a dissociation stage. Appropriate positive (*Babesia leo*, provided by Dr. B. Penhzorn, University of Pretoria, Onderstepoort, RSA) and negative controls were included in all assays. All samples were run in triplicate and threshold values averaged. Assay efficiency is 93.2%.

Because *Babesia sp.* do not have a white blood cell stage, it was biologically relevant to determine relative hemoparasitemia in relation to RBC concentration. As mammalian RBC do not contain nuclei, genes typically used to normalize between samples for quantitative PCR (such as GAPDH) could not be used. Archived blood samples had been frozen, so RBC were lysed and RBC concentrations could not be determined. Therefore, samples were normalized using hemoglobin concentrations (Pentra 60, Horiba ABX Montpellier, France) as a proxy for RBC number. Quantification of hemoglobin in frozen whole blood and red blood cell pellets were shown to correlate with hemoglobin concentrations in fresh whole canine blood (data not shown). Relative quantity of hemoparasite DNA was then calculated as average threshold cycle normalized by hemoglobin concentration and expressed as the fold difference greater than the sample with the smallest quantity of hemoparasite DNA.

### Density gel gradient electrophoresis (DGGE)

To determine if more than one type of hemoparasite was present within individual blood samples, a 477 bp fragment of the 18 s rRNA gene was amplified by PCR using primers (Bab6: 5′ GAG TAG/T CAA TTG GA G 3′ and Bab7: 5′ GGC AAA TGC TTT CGC AGT AG 3′) with a GC-clamp (CGCCCGCCGCGCCCCGCGCCCGGCCCGCCGCCGCCGC-) attached to the 5′ end of the forward primer to improve separation of closely related sequences. DNA extracts were thawed, and 2.5 µl was added to a 25 µl reaction volume containing standard amounts of GeneAmp reagents (Applied Biosystems, Foster City CA, USA), 2.5 pmol of each primer and 1.5 mM MgCl_2_. Amplification (94°C for 2 min; 35 cycles of 94°C for 1 min, 55°C for 1 min, and 72°C for 1 min; and 72°C for 5 min) was performed in a thermocycler (Applied Biosystems model 9700). Amplified products were separated and visualized on a 12% polyacrilamide gel with a 0–40% urea-formamide gradient (Dcode Electrophoresis Reagent Kit, BioRad, Richmond, CA, USA) run in TAE buffer at 60°C and 300V for 4 ½ hours in a BIORAD D-GENE apparatus (BioRad, Richmond, CA, USA). Positive controls (pure isolates of *Babesia leo*, *B. canis*, *B. microti and Cytauxzoon felis* donated by Dr. Patricia Conrad, University of California, Davis, CA, USA), as well as no DNA-negative controls were included with each electrophoresis. DNA was stained with a fluorescent nucleic acid stain (GelStar Nucleic Acid Gel Stain, Cambrex, Rockland ME, USA) and examined under ultraviolet light. Seventy-four representative bands from 31 lions were sequenced by cutting the band out of the gel and soaking in dH2O overnight, re-amplifying the DNA from the band using a new forward primer (Bab8: 5′ATT GGA GGG CAA GTC TGG 3′) with Bab7 primer (459 bp). Amplicons were purified (ExoSAP-IT, USB Corporation, Cleveland OH, USA) and nucleotide sequences of both strands were determined using an automated capillary sequencer (Applied Biosystems 3730XL, Foster City CA, USA) at the University of Chicago Cancer Research Center DNA Sequencing Facility. Sequences were compared to published sequences of confirmed strains in the GenBank database to confirm their identity.

### Hepatozoon sp. and Mycoplasma sp. real-time PCR

To determine the extent to which total hemoparasite loads were due to *Hepatozoon sp.*, a quantitative real-time PCR assay was developed to amplify *Hepatozoon sp.*, because an appropriate real-time PCR primer specific for all *Babesia spp.* that did not cross-react with other hemoparasites could not be designed. PCR conditions were the same as for the generic hemoparasite PCR using forward (Hep1: 5′ GGG ATT AGG GTT CGA TTC CG 3′) and reverse (Hep2: 5′ CCT CTC TTA TGC TGT TAG AAT TGG 3′) primers. Blood samples with a total fold difference greater than 10 that also had *Hepatozoon sp.* identified within the sample by DGGE were quantified using this PCR. Amplification conditions were 94°C 5 min; 40 cycles of 94°C for 2 min, 55°C for 30 s, 72°C for 30 s; and a dissociation stage. Positive controls were included with each plate to standardize across reactions, and no DNA-negative controls were included in all assays. All samples were run in triplicate and threshold values averaged. Assay efficiency was 96.3%.

Relative quantities of *Mycoplasma sp.* were calculated using previously published methods[26] for a subset of samples representing 10 animals with high fold differences (>10) sampled during an outbreak with mortality, 10 animals with low fold differences (<10) sampled during an outbreak, and 10 randomly selected samples from outside the outbreaks.

### Bone marrow fat quantification

Percent bone marrow fat is directly related to the percent dry weight of marrow measured from earlier calibrations using Soxhlet fat extraction. Long bone marrow samples were collected, dried, and the % marrow fat calculated by this method. The details of these techniques are provided in Sinclair & Arcese [27].
